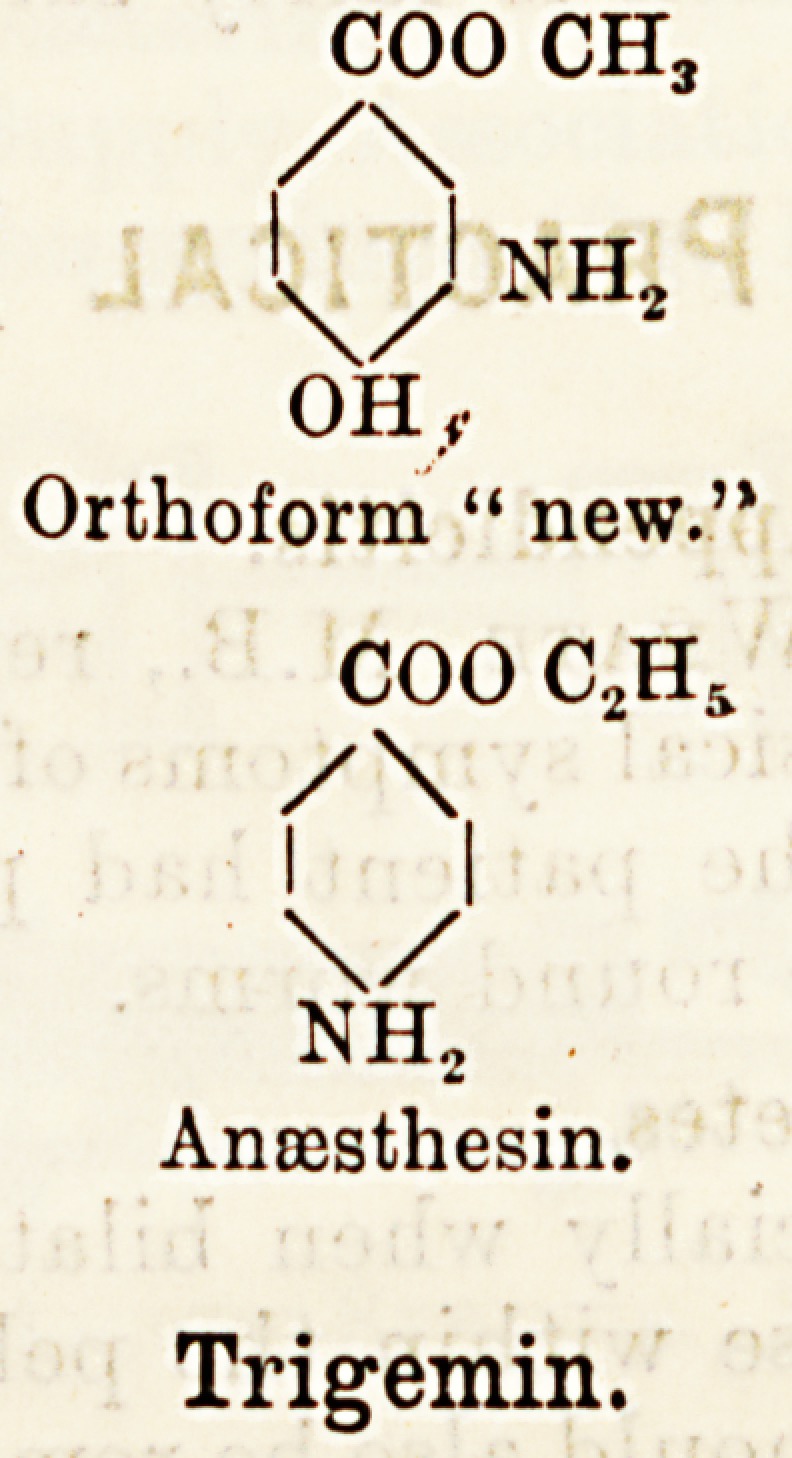# Remedies and Their Uses

**Published:** 1906-10-06

**Authors:** 


### Remedies and their Uses.

### Anæsthesin.

Anæsthesin is a fine white crystalline powder, practically insoluble even in hot water, but easily soluble in alcohol and other substances, including oils. In almond oil it is soluble to the extent of 2 per cent., in olive oil up to 3 per cent., and this solution can be sterilised by heat without decomposition. Its action is indicated by its trade name, and it may be employed both internally and externally to relieve pain. Internally it has been successfully employed to relieve the pain of gastric ulcer and gastralgia, and for this purpose it should be given in doses of about five grains two or three times daily ten to fifteen minutes before food. It is best taken as a powder. In whooping-cough and other paroxysmal affections anæsthesin may be ordered as a lozenge, its action being similar to that of cocaine and other cough-lozenges, only of longer duration. It is, moreover, very useful in tuberculous laryngitis; it may be used as a spray (3 per cent. solution in 45 per cent. alcohol) or, as advised by Von Noorden, as a powder for insufflation. Five grains in twenty grains of oil of theobromine form a useful suppository in cases of hæmorrhoids. For various forms of eczema and pruritus a 5 per cent. to 10 per cent. ointment with lanoline or vaseline bases may be employed; while painful ulcers, burns, and granulating surfaces may be treated with a dusting powder containing equal parts of anæsthesin and dermatol, or anæsthesin, dermatol, and talc powder. For destroying the nerve in a painful carious tooth a paste may be made thus:—

| | |
|---|---|
| Acidi arseniosi ... ... ... ... | 4 parts |
| Anæsthesini ... ... ... ... | 2 parts |
| Ol. caryophylli ... ... ... ... | 1 part |
| Creosoti ... ... ... ... ... | q.s. |

For urethritis, either infective or due to mechanical irritation, bougies containing seven or eight grains of anæsthesin will be found valuable in diminishing pain.

It has no irritating effect on mucous membranes or on the hard surfaces of wounds; it may be conveniently employed as a dusting powder previous to scrapings, curettings, or the application of caustics, in order to induce local anæsthesia. A 20 per cent. to 40 per cent. solution in alcohol is employed by Haig for instilling into the ear in painful conditions. The solution is mixed with a solution of thymol 2 per cent. in glycerin, five parts of the anæsthesin solution being added to forty-five parts of the thymol solution.

Chemically, anæsthesin closely resembles orthoform "new." It is, that is to say, a benzoic acid derivative, but it contains no hydroxyl group, as does orthoform. The hydroxyl group is thought to be responsible for certain unpleasant by-effects noted in specially susceptible individuals after the external application of orthoform "new." The difference in composition is seen when the following formulæ are compared:—

Orthoform "new."

Anæsthesin.

### Trigemin.

This compound is a drug for internal administration, the action of which is limited to the diminution of pain. It is not a hypnotic, it is merely an analgesic; and, in spite of its mild action, it is said seldom to fail to relieve pain. It has a further advantage in not depressing the heart. It appears to have a special action on the fifth cranial nerve. Overlach (B.K.W., 1903, No. 35), who tried trigemin in fourteen cases of neuralgia of the fifth nerve, found that after one dose of seven and a half to fifteen grains the pain was rapidly relieved for twenty-four hours or more. In first attacks the pain is often permanently cured.

In cases of generalised headache and in migraine and in the headaches of influenza and other acute fevers trigemin has been found successful, and a case of toothache in which periostitis was present has also been relieved by the drug. It has no irritating action on the stomach, and may be used to relieve the headache of acute alcoholic poisoning. Trigemin is a white slightly hygroscopic powder, not very soluble in water, and best given in a cachet. The dose is about five to twenty grains; usually a medium amount (ten grains) once, or, if necessary, thrice daily is given. The composition of trigemin is very interesting. Long heating with dilute acids decomposes it into butyl chloral hydrate and diethyl amido-antipyrin. The former drug, while exercising a specific action on the fifth nerve, lowers the blood-pressure owing to its depressant character. Diethyl amido-antipyrin, however, better known as pyramidon, is a drug which raises the blood-pressure and thus antagonises the butyl chloral effect.

Trigemin is prepared by Meister Lucius and Brünig, of Hoechst-au-Main.

## Figures and Tables

**Figure f1:**